# Compression in cultural evolution: Homogeneity and structure in the emergence and evolution of a large-scale online collaborative art project

**DOI:** 10.1371/journal.pone.0202019

**Published:** 2018-09-05

**Authors:** Thomas F. Müller, James Winters

**Affiliations:** Minds and Traditions Research Group, Max Planck Institute for the Science of Human History, Jena, Germany; University of Exeter, UNITED KINGDOM

## Abstract

Cultural evolutionary theory provides a framework for explaining change in population-level distributions. A consistent finding in the literature is that multiple transmission episodes shape a distribution of cultural traits to become more compressible, i.e., a set of derived traits are more compressed than their ancestral forms. Importantly, this amplification of compressible patterns can become manifest in two ways, either via the homogenisation of variation or through the organisation of variation into structured and specialised patterns. Using a novel, large-scale dataset from Reddit Place, an online collaborative art project, we investigate the emergence and evolution of compressible patterns on a 1000x1000 pixel canvas. Here, all Reddit users could select a coloured pixel, place it on the canvas, and then wait for a fixed period before placing another pixel. By analysing all 16.5 million pixel placements by over 1 million individuals, we found that compression follows a quadratic trajectory through time. From a non-structured state, where individual artworks exist relatively independently from one another, Place gradually transitions to a structured state where pixel placements form specialised, interdependent patterns.

## Introduction

Explaining population-level distributions of cultural traits across both spatial and temporal dimensions is a central goal of cultural evolutionary theory [1–10]. Gaining traction within this framework is the idea that cultural traditions organise themselves into compressible patterns in response to simplifying pressures [3, 11–20]. Compressible patterns are found in many cultural domains, from language and music to technology and art [15, 21, 22]. What makes a set of cultural traits compressible is if the length of the set is describable by a rule shorter than simply listing each individual trait. Islamic geometric art, for instance, is highly compressible in that many artworks within this tradition are built on simple, generalisable rules, involving the repeated use of basic shapes to generate novel, open-ended patterns [23]. Increasing compression is therefore characterised by the organisation of cultural information into more predictable patterns; the less irreducible unpredictability remaining in the data, the greater the amount of compression [19].

Compressible patterns can become manifest in a distribution of cultural traits via two processes. The first is through spreading homogeneity in a population of cultural traits [24]. Consensus formation is an example where, through the differential amplification of behaviours, variation is regularised via the spreading of homogeneity [25]. The second is to organise variation into structured, interdependent patterns [20, 26]. Heraldry is structured because it can generate novel designs by using underlying rules to recombine a finite set of components (e.g., motifs combine with tinctures of metals, colours, and furs to create a design) [27]. The main difference between the two is that the first type of compression removes variation and encourages homogeneity, whereas the second type of compression maintains variation and imposes structure.

Using a novel, large-scale dataset from *Reddit Place*, a collaborative pixel art project involving over one million participants, we investigate the emergence and evolution of compressible patterns on an unprecedented scale. Our general hypothesis is that compressible patterns are present in Place and driven by artworks being organised into structured, interdependent arrangements. By measuring compression, and controlling for changes in the frequency distribution of variation, we discriminate between homogenising and structuring processes. If changes in variation are driving compression, then decreases in variation should result in increases in compression (i.e., homogeneity spreads). Conversely, if variation is being organised into structured and specialised patterns, then increases in compression are decoupled to some extent from changes in the frequency distribution of variation (i.e., structure spreads).

### From pixels to artworks: Reddit Place

**Fig 1:**
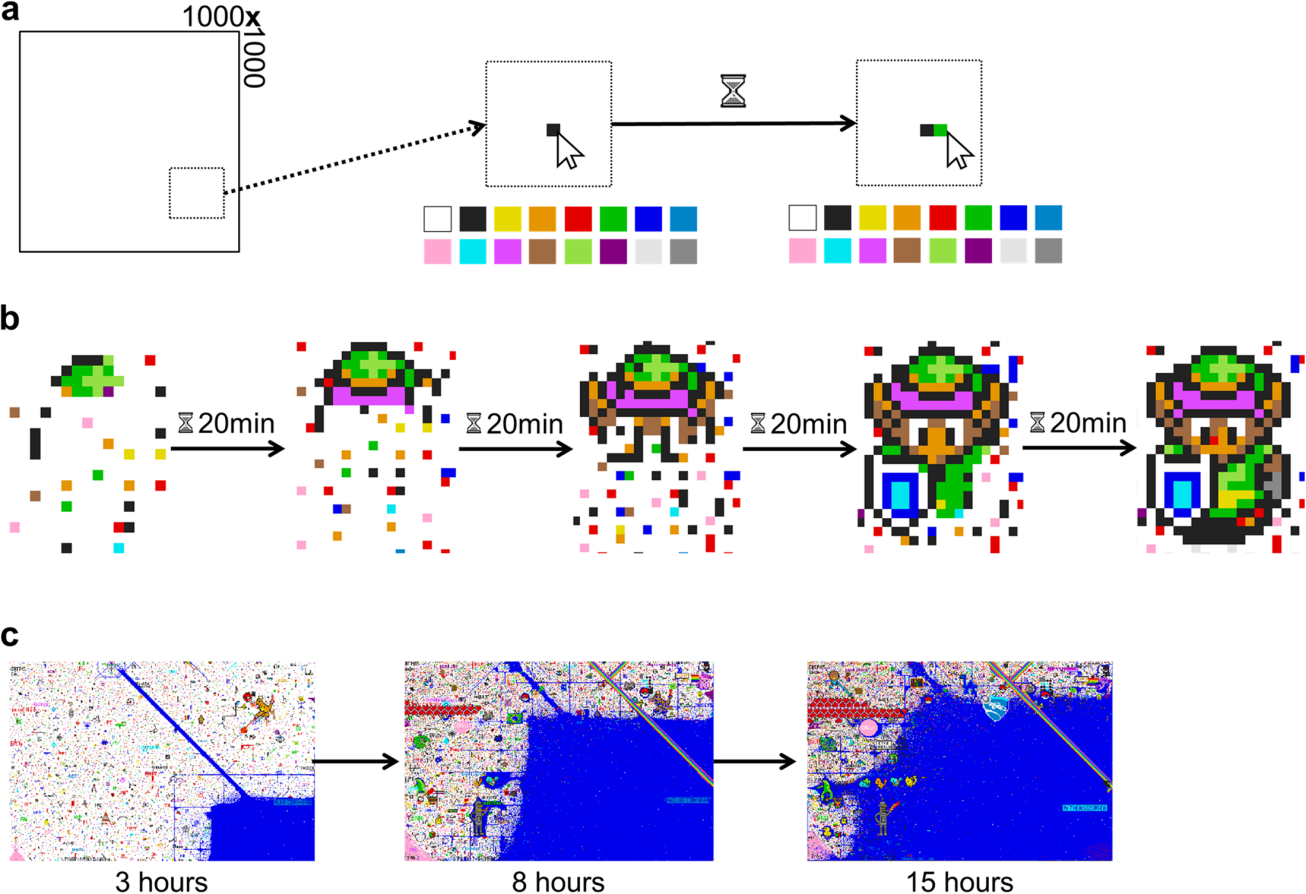
The basic mechanisms of Place. a) Reddit users could select a single pixel from a set of 16 colours, place it anywhere on the 1000x1000 canvas, and then wait for a fixed period until they could place another pixel. This represents the introduction of variation to a homogeneous space on an individual level. b) Through collaboration, simple artworks can form, with variation being organised into structured, compressible patterns. c) Some groups pursued goals internal to Place, like dominating as much of the canvas as possible, which highlights how compressible patterns can arise via the removal of variation.

For 3 consecutive days (72 hours), starting from March 31st 2017, Reddit opened a white 1000x1000 pixel canvas to its users. When visiting the page, all users that had been registered prior to the start of this event were allowed to place pixels on this canvas (see Fig 1a for details). This was accompanied only by the following developer post:

There is an empty canvas. You may place a tile upon it, but you must wait to place another. Individually you can create something. Together you can create something more.

Crucially, after Reddit users had placed one pixel anywhere on the canvas, a fixed period (initially 5 minutes) was imposed before a user could place another pixel. Thus the *collaborative* aspect of the project: On their own, users were severely limited with regard to their possible creations. Because of this, it is of no surprise that cooperation was present from early on, even though many initial pixel placements were disorganized, chaotic and lacking in overall direction. Through these collaborations, taking place via posts on Reddit, simple artworks began to form (Fig 1b), corresponding to one way of organising variation into structured patterns. Creations covered a wide range of themes, from national flags and sports teams to video games and general *geek culture*, with some instances being geared toward goals internal to Place. For example, members of *Blue Corner* tried to dominate as much space as possible by repeatedly placing blue pixels in non-blue regions (Fig 1c).

### Cultural evolution in place

Culture exists as information in the minds of individuals (e.g., an idea for an artwork or artistic techniques) and is expressed in a population as cultural traits via observable behaviours (e.g., the act of placing paint on a canvas) as well as tangible artefacts (e.g., artworks) [28, 29]. Place meets all the necessary prerequisites of a cultural evolutionary process: there is reproduction, variation, and change in a population of cultural traits [30]. Artworks form lineages of descent, coloured pixels constitute cultural traits, and both deterministic and stochastic factors can govern the rise and fall in the frequency of these traits.

The core mechanism of Place is relatively simple, as it involves choosing and placing a coloured pixel on a canvas. This process fulfils the minimal requirements of cultural transmission through the repeated observation and production of behaviours [31]: Individual users are able to observe the behaviour of others via previous pixel placements, and to then use these observations to update their beliefs and make a decision about which coloured pixel to choose and where to place it on the canvas. What makes Place unique in this respect is that the transmission dynamics and population structure are far more complicated than standard cultural evolution experiments [15] and simulations [32]: Individuals are free to organise themselves into groups, and the sheer number of individuals maps more closely to the scale of transmission events and group structures we observe in modern, large-scale societies.

The nature and scale of the task highlights several additional differences between Place and standard cultural transmission experiments [33]. First, there is perfect retention of pixel placements between time-steps, removing the need to remember or reproduce pixel placements at a previous time-step. Second, due to the complexity of the transmission dynamics, discerning generations is not straightforward at the level of users. A single individual can appear at many different time steps, meaning there are several possible transmission patterns (e.g., one-to-many, many-to-many, oblique and horizontal transmissions; see [33] for overview). Lastly, users were not provided with an overt goal in choosing and placing coloured pixels. Instead, the motivation for creating and participating in artworks was determined by the preferences of individuals. Compare this to most experimental tasks where participants are generally provided with a training regime and given explicit instructions about the goal (e.g., to successfully communicate [34] or asked to memorise a set of sequences [15]).

**Fig 2:**
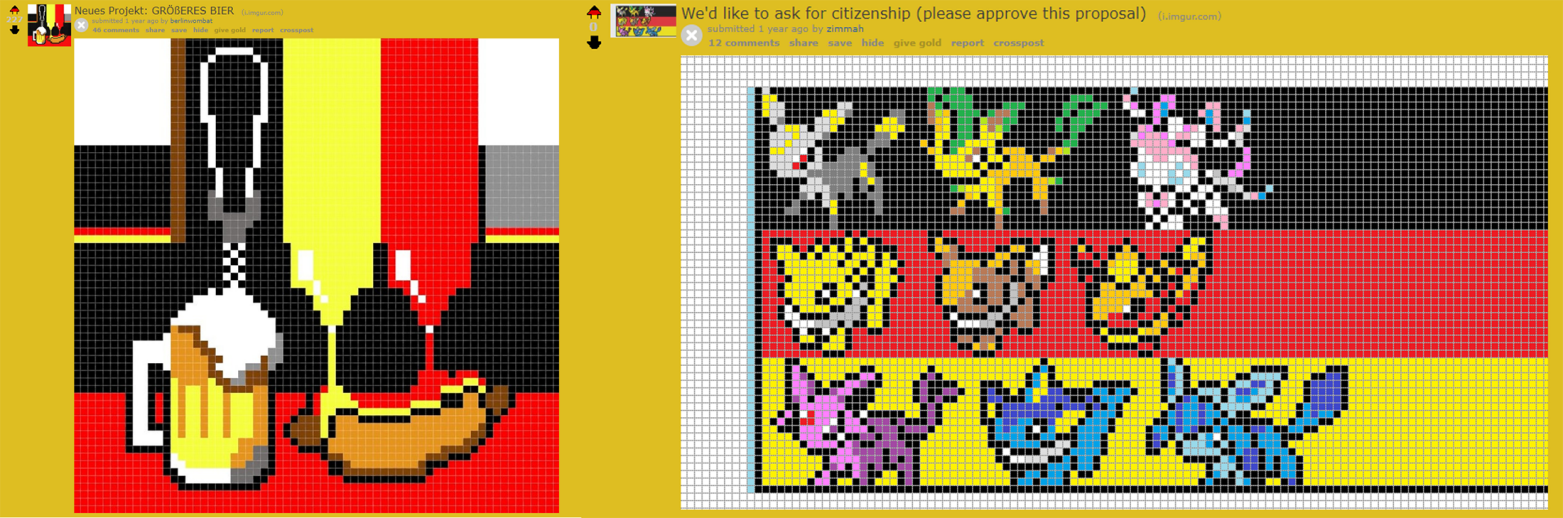
Two proposals for artworks in PlaceDE subreddit. Left: Proposal for artwork which successfully made it onto the canvas with 225 votes. Right: An unsuccessful proposal with 0 votes. Subreddits allows for selection of artworks based on up-voting or down-voting as well as via comments below the proposals.

Overlaid on top of the task itself are the subreddit communities. Users could come together to strategically plan pixel placements and organise the resources of their respective subreddits in maintaining, expanding, and sabotaging artworks. As such, Place captures individual variability of users as well as social factors emerging from within-group goals (e.g., to mark group identity). An example of this is found in the *PlaceDE* subreddit (see Fig 2): users often proposed new pixel art projects to the community, which were then subject to discussion and voting. This process played a within-group role for determining which artworks eventually made it onto the canvas. So, even before users placed a pixel, there were cultural evolutionary dynamics governing the selection of ideas for artworks.

In many respects, Place forms a microcosm of cultural evolution in a similar manner to Petri dish bacteria in biological evolution [35]. It is also worth noting that the dissimilarities between Place and other cultural phenomena are not as great as their similarities in terms of evolutionary dynamics. Just as *e.coli* differ from humans in that they can reproduce asexually and transmit genetic information horizontally as well as vertically [36], so too does Place differ from other, well-studied examples of cultural evolution (e.g., language, technology etc).

### Research questions, hypotheses and predictions

Any evolutionary system where resources are finite requires solving the problem of competition and coexistence [36, 37]. Like in the biological domain, cultural traits (i.e., coloured pixels) that enter into competition with one another cannot coexist indefinitely (see *competitive exclusion principle*; [38, 39]): Either one trait drives the other to extinction or competing traits come to occupy distinct niches (see *niche differentiation*; [40, 41]). Competition resulting in extinction is essentially a homogenising process (as highlighted by cases like the Blue Corner; Fig 1c) and niche differentiation maintains and organises variation by structuring it into specialised patterns (e.g., Fig 1b). What remains unknown is the extent to which these processes shape the overall distributional patterns of the canvas (i.e., the complete set of pixel placements). Specifically, we ask: (a) Does compression follow a predictable time course? (b) Is this overall compression driven by variation being reduced or structured? (c) Does the canvas become increasingly stable?

Place provides a particularly apt and novel dataset for answering questions pertaining to cultural evolution. A central feature of Place is its fixed, bounded space of 1000x1000 pixels, placing a hard constraint on the maximum amount of variation in a population (i.e., exactly a million pixels). Having a finite space allows us to investigate whether the density of the canvas interacts with evolutionary dynamics in shaping the distribution of pixel placements. This leads us to two predictions about the time course progression of Place:

- When Place is sparsely populated with artworks, the canvas will decrease in compression, have low stability, and increase in variation.
- When Place is densely populated with artworks, the canvas will increase in compression, have higher stability, and plateau in variation.

Initially, users solve the competition-coexistence problem by creating different artworks in unused regions of the canvas, offsetting competition between artworks of different groups. Such dynamics are even formally codified in some cases, exemplified by rule 1 of the *Green Lattice* subreddit: Protect art, do not destroy it. Work around existing pixel art. Any new art must be OUTSIDE our borders unless we approve it first. The overall effect of this strategy results in the canvas becoming less compressed over time as distinct groups create artworks in unused regions. Growth in diversity should also be reflected in a more uniform distribution of coloured pixels and lower levels of stability.

The viability of such a solution depends on there being a large number of unused regions to mitigate between artwork competition and for innovations to promulgate. This is not the case at the latter stages of Place. Now, the canvas is densely saturated with artworks, making the number of unused regions a rarity and increasing the probability of competition. Compressible patterns are advantageous because these are easier to produce, maintain, and generalise. If two artworks compete for pixel placements, then the more compressible of the two is better adapted to this niche as it can expand quicker and is more readily reconstructed than its less compressible counterpart.

Group structure acts as a countervailing force to an increased pressure for compressible artworks (perhaps in an analogous fashion to the role of communication in language evolution [16, 20]). First, each group has its own internal goals for creating artworks, and it becomes increasingly costly for one group to dominate the canvas. Even with a maximally homogeneous artwork, maintaining and expanding an artwork at the expense of other artworks requires a significant number of users (relative to the total population). Second, by modifying pre-existing artworks to create derivative innovations, groups can mitigate competition by preserving elements of old artworks whilst facilitating the creation of new ones. This second point explains why we predict structured, stable, and diverse patterns to emerge: the specialisation and integration of artworks injects structure into the canvas via the creation of shared features.

## Materials and methods

### Procedure

When placing a pixel on the canvas, Reddit users could navigate and zoom into areas of interest to allow for greater precision (especially important for mobile devices) and overview (for the technical details on how Place was programmed, see https://redditblog.com/2017/04/13/how-we-built-rplace/). As mentioned in the introduction, one crucial feature of the project was that a waiting period was enforced after a pixel had been placed. This period had initially been set to 5 minutes, but was changed by the administrators several times while the canvas was live to accommodate the incoming traffic on Reddit’s web page. The creators of Place estimate that approximately 80,000 users were connected to the canvas simultaneously at peak time. In the total runtime of 72 hours, about 16.5 million pixels were placed by the Reddit community, corresponding to approximately 1.2 million active unique users. Some caution is warranted, however, with this number of users, since Place was deliberately designed to also allow the programming of bots. Still, users had to adhere to the waiting period imposed by the rules of the project and stick to their account registered prior to the start of Place, irrespective of whether they placed the pixels manually or automatically. The only exception to this was a minority of users who discovered a loophole that allowed for multiple pixel placements at once if they were sent to the client at the exact same time; however, this exploit was only used to change around 15,000 pixels, i.e. roughly 0.09% of the total placements (https://redditblog.com/2017/04/13/how-we-built-rplace/). All in all, we do not see any of the issues described as problematic for our data, since they mean that some individuals were able to have more impact than others (through technological means), while the general notion of Place as a model for cultural evolution still holds.

### Data

**Fig 3:**
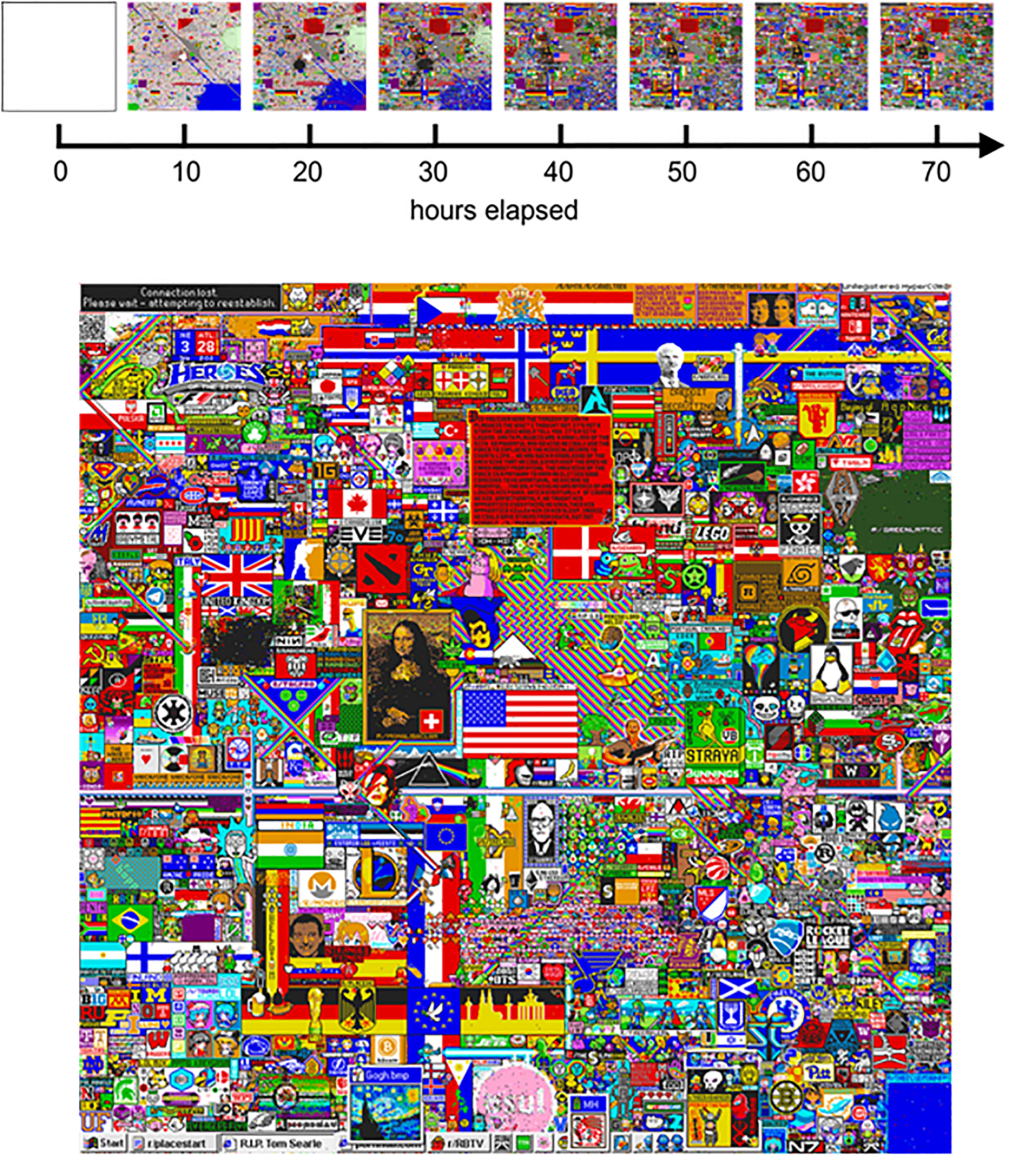
The development of the place canvas. Top: State of the canvas in 10-hour-intervals. Bottom: The final image after 72 hours, frozen in its state.

The raw data of Place has been made freely available online by the Reddit administration: https://www.reddit.com/r/redditdata/comments/6640ru/place_datasets_april_fools_2017/. However, as of 2nd August 2018, the link to this file is not working properly (see Data Availability below for how it can be accessed on the OSF). In it, every pixel placement is provided with the corresponding colour chosen, user id, and a time stamp. The time stamps exhibit a resolution of one second. Since the full data also includes a period before the project went live, during which the creators of Place conducted some final tests, we reduced this raw file to a runtime of exactly 72 hours by cutting off this testing period. From the resulting dataset, we prepared our analysis by constructing a separate bitmap image (.bmp) for each unique time step showing the current state of the canvas. This resulted in 259,194 images to work from. Fig 3 gives examples of the canvas for every 10 hours in the time course of the project, as well as showcasing the final outcome of Place. When the 72 hours had elapsed, the canvas was frozen in this state, and Reddit users were aware of this fact for the duration of the project.

### Measures

#### Compression

**Fig 4:**
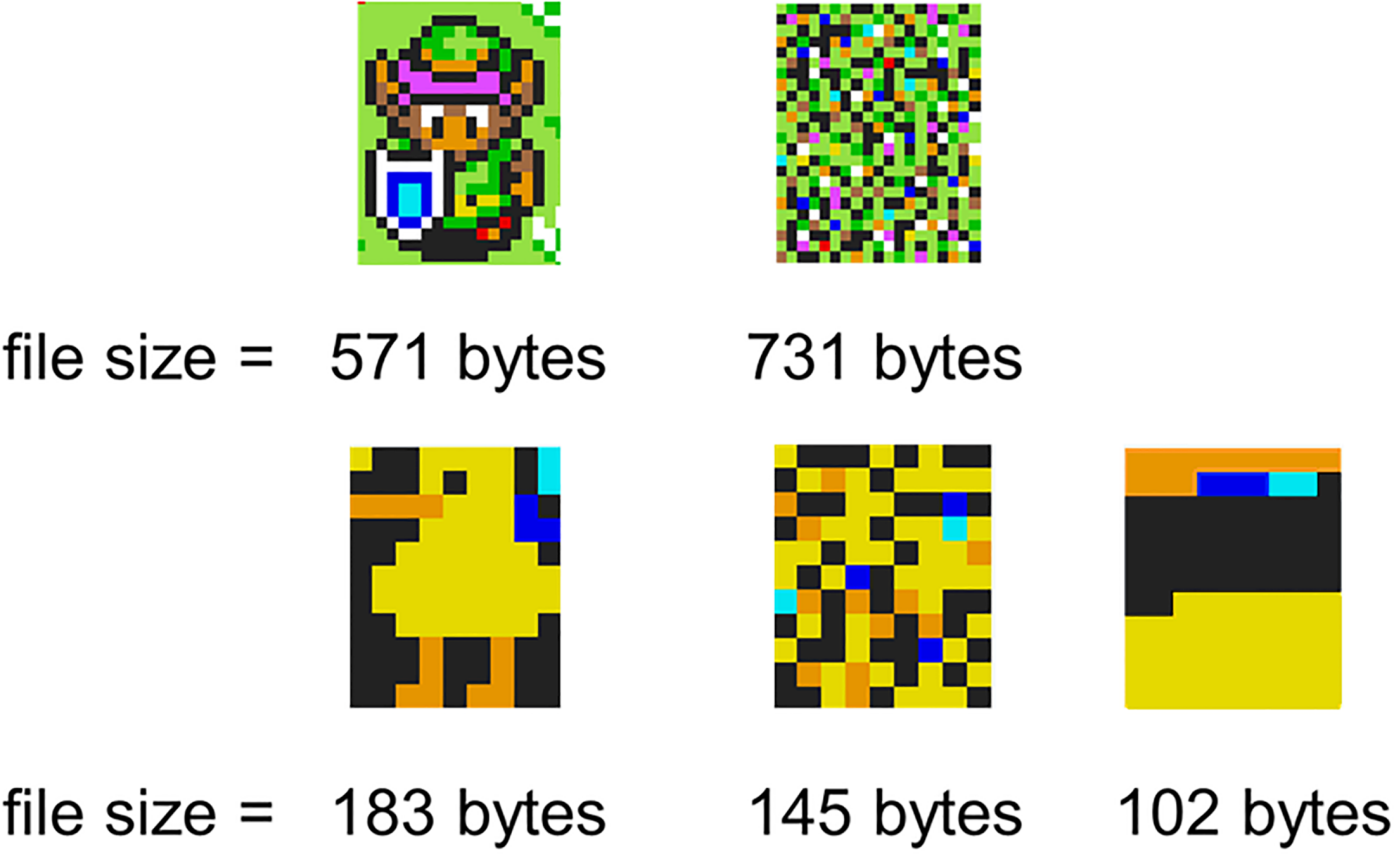
Compression measure applied to an image. Top: How a .png format can be used to assess the compressibility of an image: The pixels on the image to the right are identical to the left image in colour value, but shuffled randomly in their position. Still, the file sizes indicate that the left image is more compressed. Bottom: Randomisation can still create compressible patterns, as demonstrated by the image in the middle, which is more compressible than the original. Note, however, that a strongly structured image like the one on the right will always be more compressible than the average randomised image.

Compression was measured by converting all of our .bmp images to png images (.png). We used the DEFLATE compression algorithm found in the PIL package in Python to generate a set of maximally compressed .png images (for two simple examples, see Fig 4). As the images generated are always 1000x1000 pixels, we can compare the compression sizes of each image with one another based on their time stamp. In line with the original .bmp images, we obtained 259,194 images this way. The measure of compression equates to the file size of the .png images, read out automatically in Python.

There are two ways compressed patterns can emerge in Place. The first is that changes in compression correspond to changes in the frequency distribution of colour tiles (i.e., our measure of global entropy). If this is the case, compression should closely track the time course trajectory of global entropy. The second possibility is that variation is being maintained and structured into regular and predictable patterns. In this scenario, the trajectory of compression should be decoupled to some extent from changes to the frequency distribution of colours, with compression increasing relative to the amount of global entropy.

#### Global entropy

**Fig 5:**
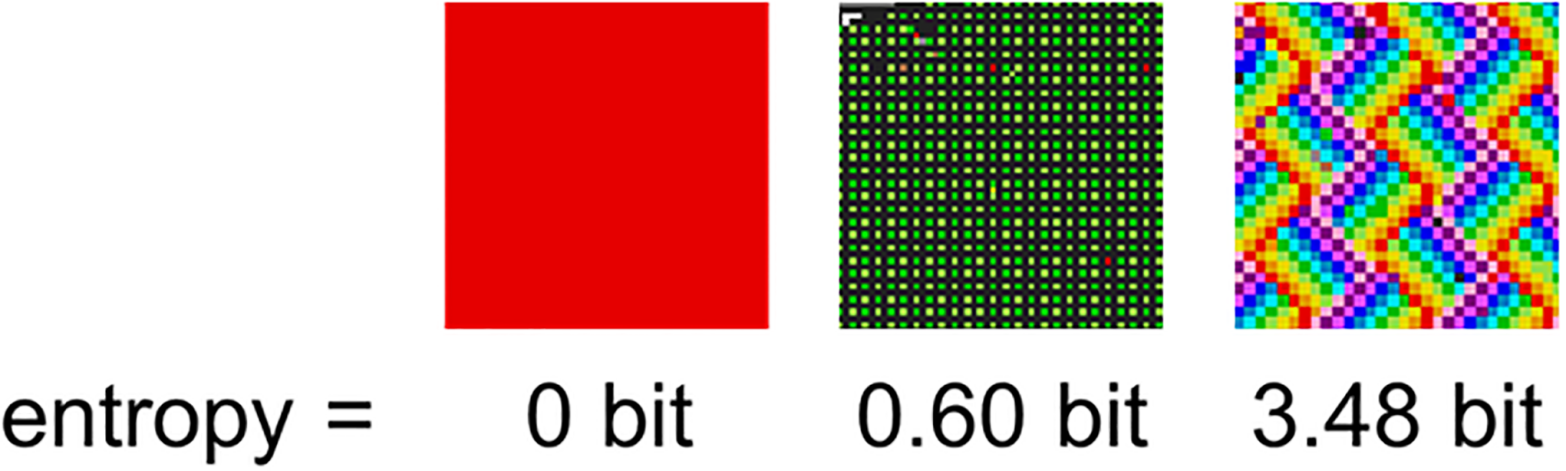
Some real examples from the canvas and their corresponding entropy values. As can be seen, a completely non-uniform distribution of colours (i.e., a single colour) results in an entropy of 0 bit, whereas a strongly uniform distribution of colours (such as a rainbow pattern) results in entropy values close to the maximum of 4 bit.

The emergence of compressible patterns is often coupled to changes in the frequency distribution of pixel placements. To measure this, we first calculated a frequency distribution for each of the 16 colours available to users on each of the image files (e.g., if Blue has a frequency of 100, then this corresponds to 100 unique pixels of Blue in Place). Next, we computed the conditional entropy [42] of colours given time-steps, *H*(*C*|*T*):

$$\begin{array}{c} H\left(C|T\right)=-\sum_{c\in C}P\left(c\right)\sum_{t\in T}P\left(t|c\right)\;log_{2}P\left(t|c\right) \end{array}$$ (1)

where *C* refers to the set of 16 colours {*c*_1_, …, *c*_16_} and *T* is the set of time-steps {*t*_1_, …, *t*_259,194_}. *H*(*C*|*T*) therefore measures the predictability of a colour at a specific time-step (i.e., the non-uniformity of the frequency distribution; see Fig 5). As there is a fixed space of 1000x1000 pixels, a maximally unpredictable state corresponds to a uniform distribution: each colour is represented at an equal frequency (62,500 pixels) and *H*(*C*|*T*) = 4 bit. A maximally predictable state is a non-uniform distribution, where *H*(*C*|*T*) = 0 bit and consists of a single colour at a frequency of 1,000,000 pixels (with the other 15 colours at a frequency of 0).

#### Local entropy

**Fig 6:**
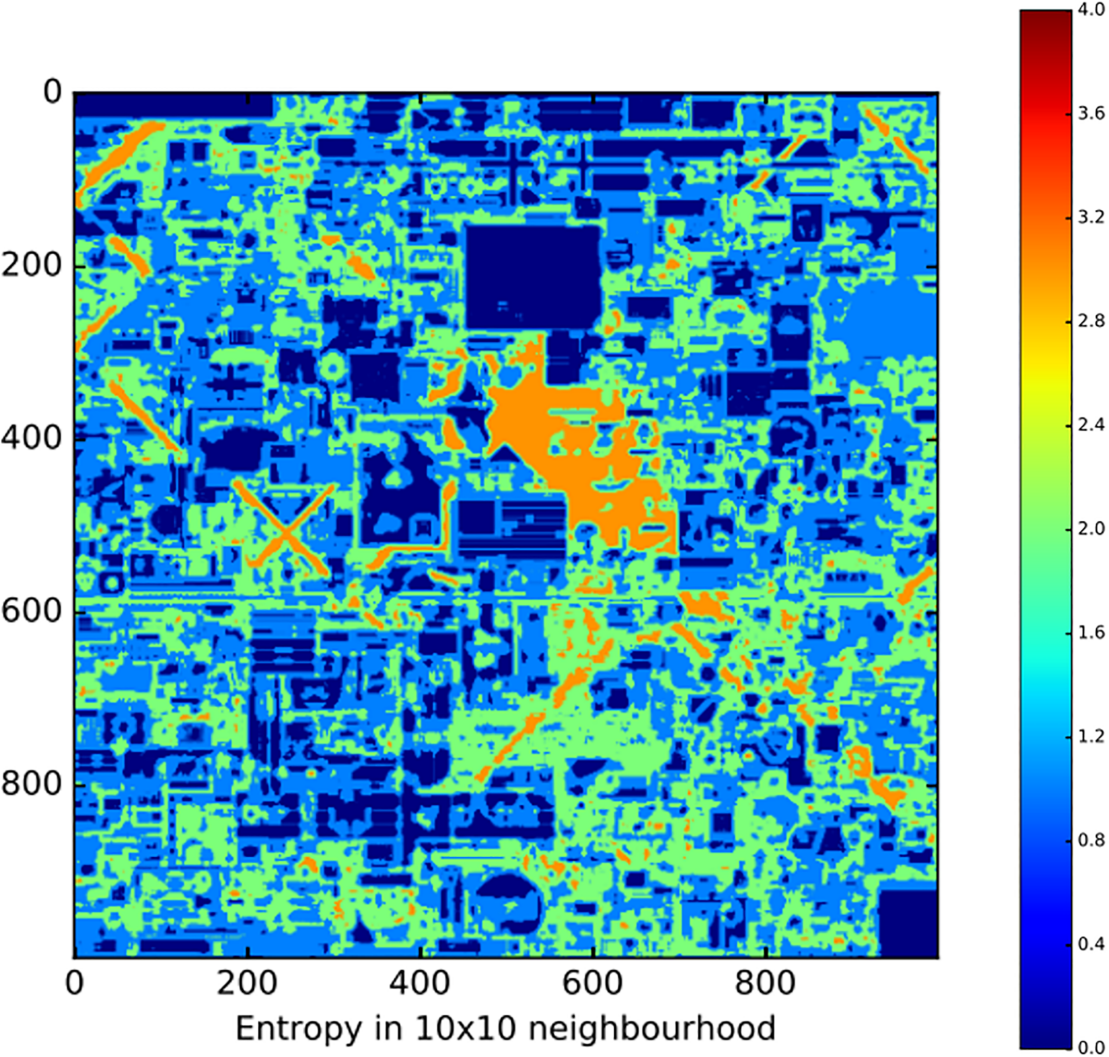
Local entropy of the final canvas. Blue regions correspond to 10x10 neighbourhoods with low entropy and orange regions correspond to 10x10 neighbourhoods with high entropy.

The local entropy between pixel placements can be measured by calculating at each pixel position (*x*, *y*) the entropy of pixel placements within a 2-dimensional region [43]. Using 10x10 regions, we flatten the 2-dimensional region into a 1-dimension array, which is then passed to the conditional entropy function:

$$\begin{array}{c} H\left(C_{R}|T\right)=-\sum_{c_{r}\in C_{R}}P\left(c_{r}\right)\sum_{t\in T}P\left(t|c_{r}\right)\;log_{2}P\left(t|c_{r}\right) \end{array}$$ (2)

where *C*_*R*_ is the set of of 16 colours within a specific 10x10 region. Due to the large amount of computation power required for 1 million pixels, we restricted our use of this measure to 1-hour-steps (72 slices). As with *global entropy*, a maximally predictable pattern has an entropy of 0 and a maximally unpredictable pattern has an entropy of 4 (see Fig 6). Importantly, homogeneous patterns have a low global entropy and a low local entropy, whereas structured patterns should have a high global entropy and a low local entropy. This is because structured patterns maintain variation (hence high global entropy), with regularities being restricted to local level patterns (hence low local entropy).

#### Stability

**Fig 7:**
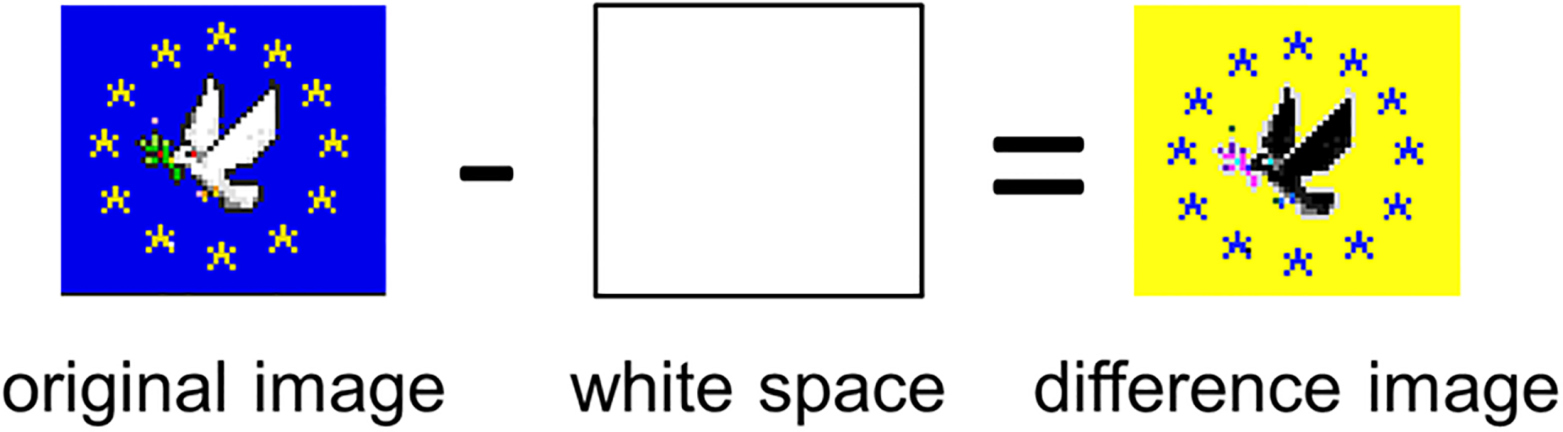
How the stability measure is applied. To compare two states of the canvas, the difference between the values of the pixels is computed. On the resulting difference image, stable pixels appear in black, since all their values are zero.

Stability was assessed by computing the pixel-by-pixel difference between different slices of the data (see Fig 7). A pixel value is seen as stable if this difference amounts to zero. The total number of stable—i.e., unchanged—pixels indicates the stability of an image. Since the resolution of 1-second-slices is too fine-grained to find meaningful development within a single time step, we ran our analyses on three different resolutions (pre-registered before analysis): a resolution of 1-hour-steps (72 slices), 2-hour-steps (36 slices), and 4-hour-steps (18 slices). Because of the way this measure is computed, coarse resolutions allow us to observe more general trends (since the amount of change between steps is larger), but these resolutions also run the risk of losing power (due to the reduction in the number of data points).

### Pre-registration

All of our predictions, with the specific time courses for the measures, have been registered on the Open Science Framework before preparing the raw data (https://osf.io/cx67g/).

## Results

### Does compression follow a predictable time course?

**Fig 8:**
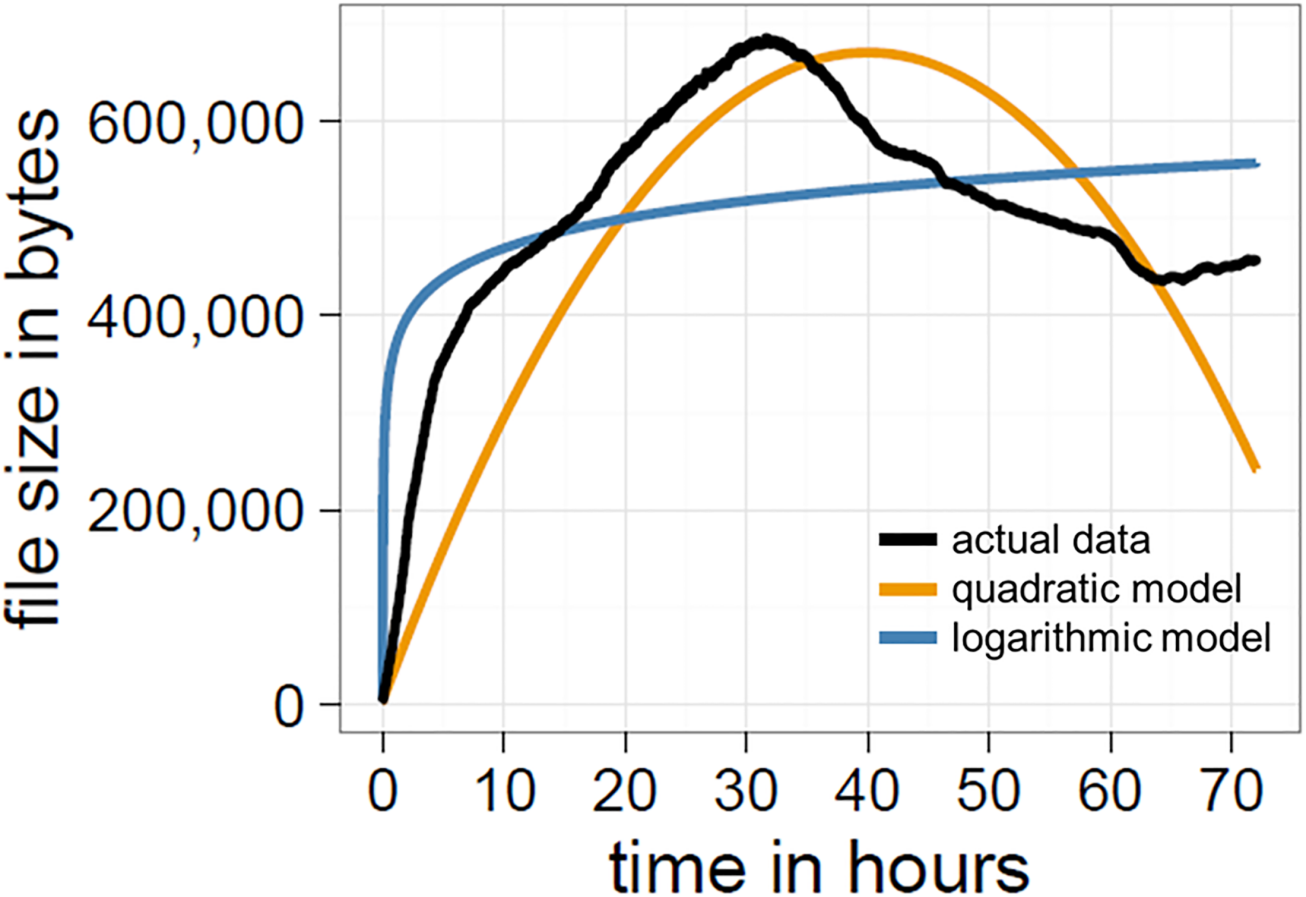
The time course of compression. File sizes in bytes are plotted in black. The quadratic and logarithmic model are plotted in orange and blue, respectively. The quadratic model showed the best fit to the data.

To test for whether compression follows a predictable time course in Place we specified three competing models: a linear model, a logarithmic model, and a quadratic model. Compressed file sizes were predicted by time only, but the models differed in the way this relation was formulated. The intercept in all models was set to 4,372 bytes, as that was the (*a priori* known) compressed file size of the initial empty canvas. After the models had been fit, they were compared using the Akaike information criterion (AIC); an estimator of the model fit, taking into account the number of parameters for a given set of data. For the linear and logarithmic models, the canvas is not predicted to become more compressed as file sizes increase monotonically. Only the quadratic model predicts higher levels of compression: file sizes initially increase, reach a peak, and then decrease at the latter stages. As predicted, we find that a quadratic model provides the best fit of the data (linear AIC: 7,199,987; logarithmic AIC: 6,701,407; quadratic AIC: 6,687,914; Δ AIC two best models: 13,493; Fig 8), supporting our contention that Place does become more compressed and follows a predictable trajectory.

One explanation for this trajectory is that the density of the canvas modulates the pressure for compressible patterns. If the spread of compressible patterns is contingent on the density of the canvas, then we predict that compression only spreads when the canvas is densely saturated with artworks. Our reasoning is that a higher density increases the probability of competition, with more compressible artworks having an advantage as these are easier to produce, maintain, and generalise than less compressible ones.

**Fig 9:**
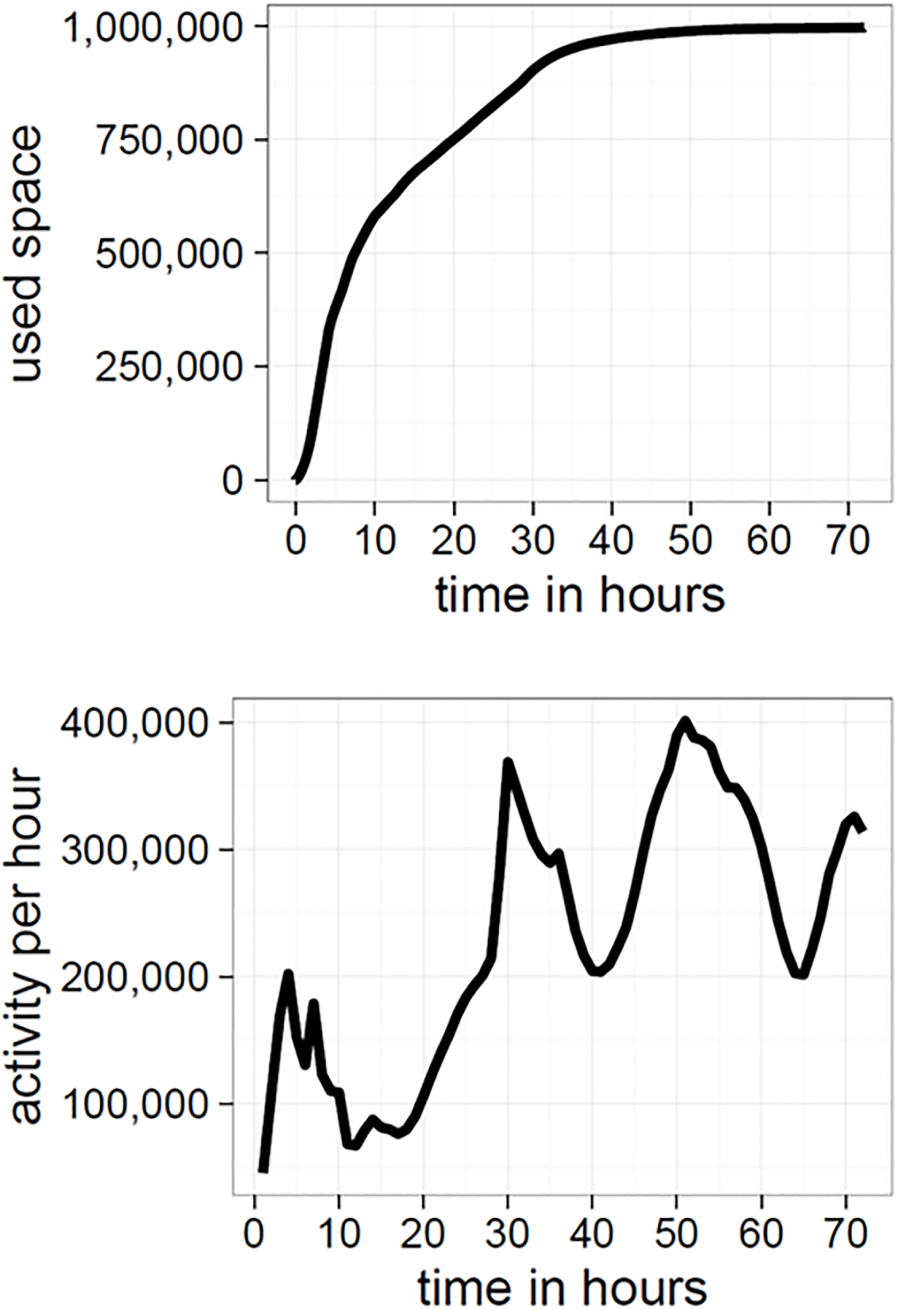
User activity and number of used pixels. Top: the amount of used space (i.e., pixels) for the duration of Place. Bottom: the amount of user activity for the duration of Place (total number of changes per hour).

To explore this hypothesis further, we constructed a more complex regression model, using the availability of space on the canvas (limited by the boundedness of Place), the activity levels of individuals, and time as a linear predictor of file size (plus associated interactions). We measured the amount of space by calculating the frequency of used pixel placements at a given time-step. A pixel qualifies as used if any user had previously placed a pixel at that particular coordinate on the canvas. As such, the number of used pixels should increase as a function of time (see Fig 9). Activity refers to the number of pixels placed on the canvas at a specific time-step (see Fig 9). Time was included to separate the variables of interest from its trajectory of a constant increase.

In isolation, more used space should increase variation on the canvas, resulting in less compressible images. However, the interaction between used space and activity captures the increased pressure for compression, resulting in a reversal of the effect (i.e., the direction of the effect changes in the interaction when compared to used pixels as a predictor). Our rationale for this is that higher rates of activity and more used space act as a proxy for increased levels of competition. The model confirms our expectation that levels of competition predict compression: the interaction between higher rates of activity and more used space is a significant predictor of decreases in file size (cf. Table 1).

**Table 1:** Results of regression model with activity, used pixels, and time (plus interactions) as predictors of compression (file sizes).

	Estimate *β*	Std. Error	t value	*p* value
(Intercept)	50,940	379	134.41	<0.001
Used pixels	0.9032	0.001345	671.51	<0.001
Activity	-123	10.07	-12.21	<0.001
Time	-7.696	0.04325	-177.92	<0.001
Used pixels:Activity	-0.002131	0.00001910	-111.57	<0.001
Used pixels:Time	0.000005548	0.00000003718	149.22	<0.001
Activity:Time	0.2103	0.0006544	321.33	<0.001
Used pixels:Activity:Time	-0.0000002009	0.0000000005795	-346.61	<0.001

### Is the trajectory of compression explained by homogenising or structuring processes?

One possibility is that the observed decrease in compression is being driven by changes to the frequency distribution of colours. A canvas with a uniform distribution (i.e., all 16 colours are represented at an equal frequency) is less compressible than a non-uniform distribution (e.g., the canvas is homogeneous and consists of a single colour). To capture changes in the frequency distribution of colours we measured the amount of global entropy. In parallel with the analysis for compression, we specified three models: a linear model, a logarithmic model, and a quadratic model. Again, all models had in common that global entropy values were predicted by time only, but differed in the way this relation was formulated. Additionally, the intercept in all models was set to zero, as it was known that the white space at the beginning would show an entropy of 0 bit. Models were then compared using the AIC.

**Fig 10:**
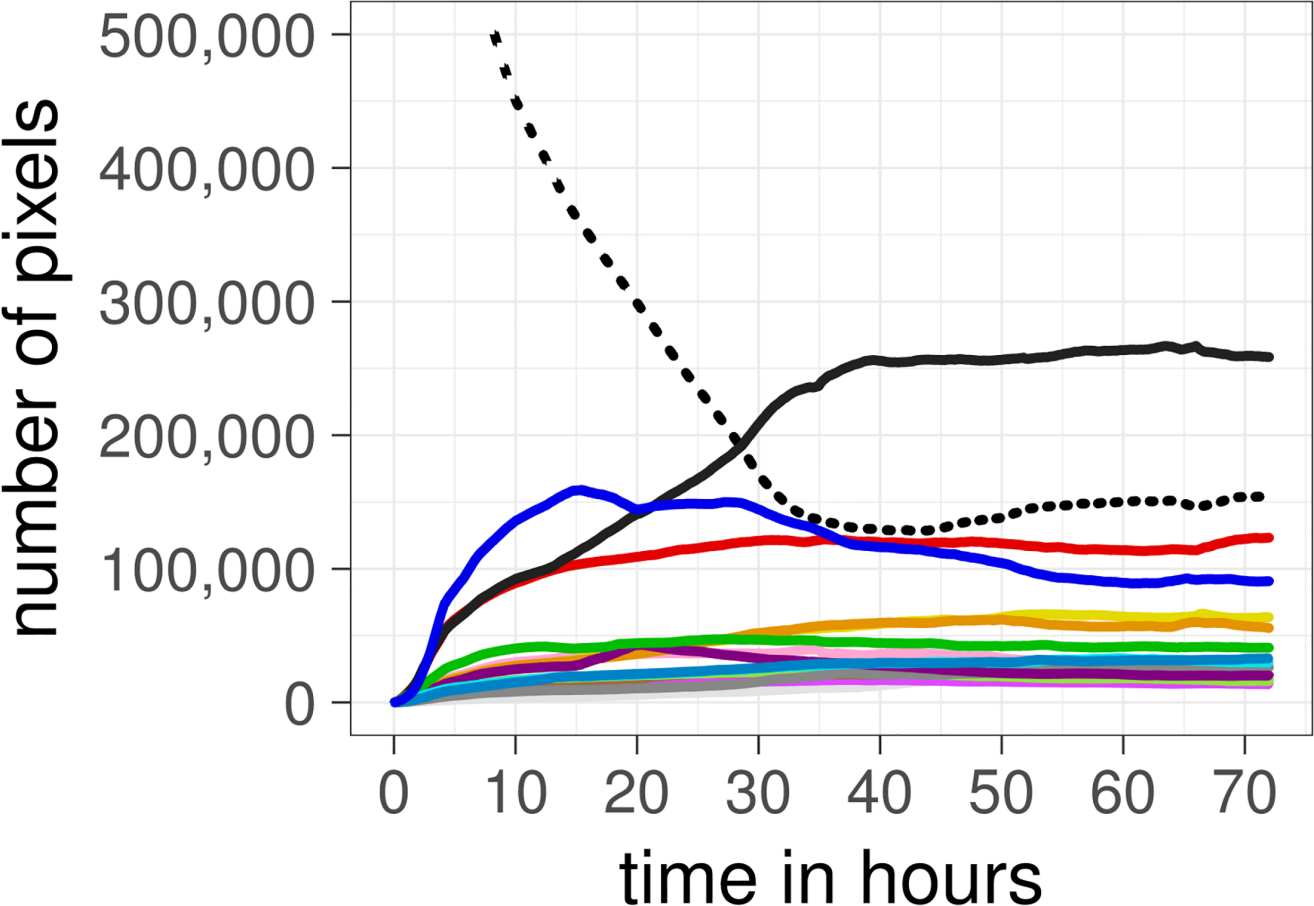
Frequency distribution of the 16 colours in Place over time. White pixels are represented by the dotted line and the remaining 15 colours are depicted in their original hue. Note that the canvas started with all of the one million pixels in white (a completely non-uniform distribution). Over time, each colour approaches its own asymptotic level and stabilises at approximately the 50-hour mark.

**Fig 11:**
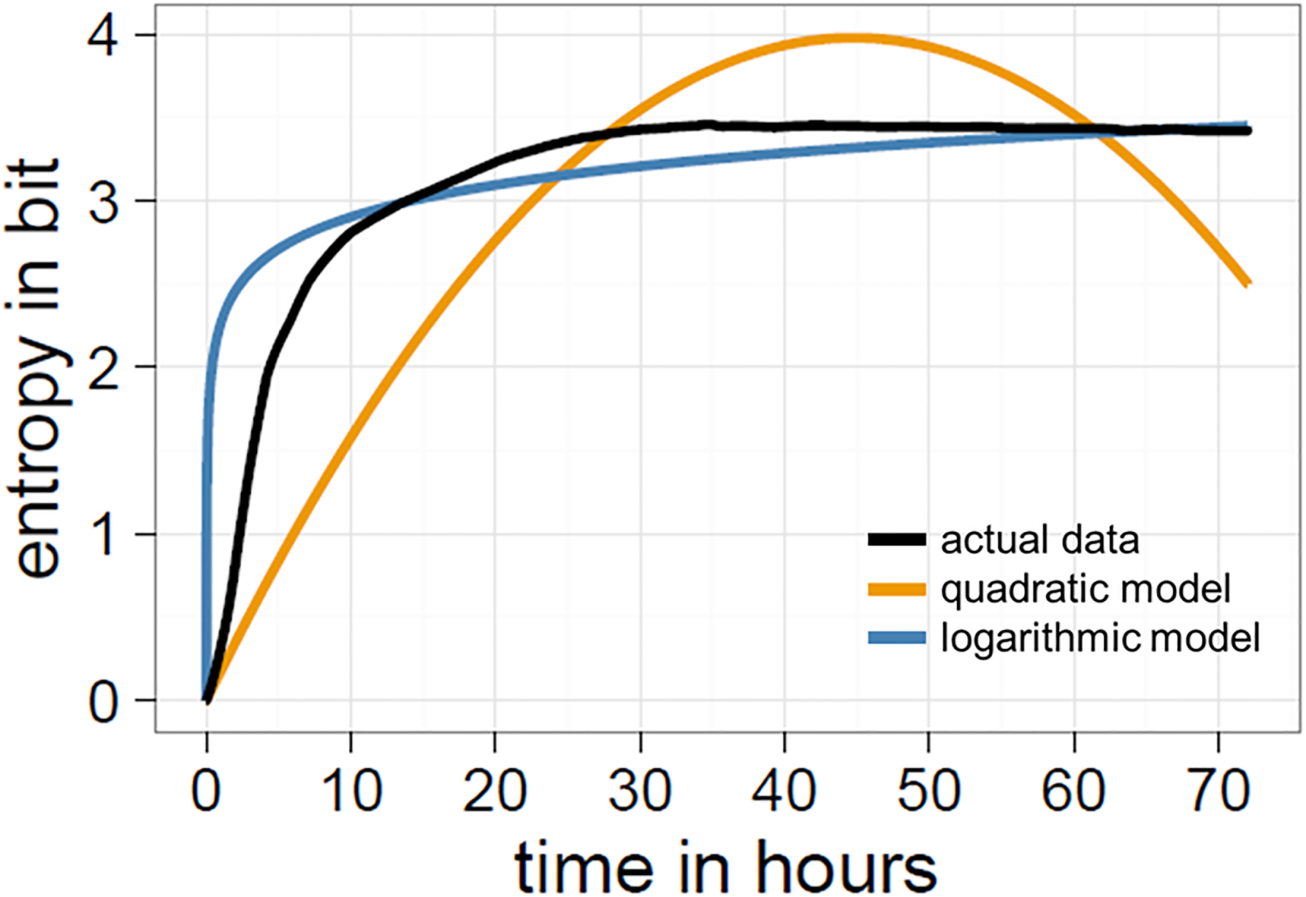
The time course of global entropy. Entropy values in bit are plotted in black. The quadratic and logarithmic model are plotted in orange and blue, respectively. The logarithmic model showed the best fit to the data.

If changes in global entropy account for changes in compression, the trajectory of this distribution should approximate a quadratic function. Instead, the distribution of colours moves away from the non-uniform distribution at the start (see Fig 10) and approaches an asymptote of approximately 3.4 bit, with a logarithmic model providing the best fit of the data (linear AIC: 874,314; logarithmic AIC: 264,064; quadratic AIC: 485,931; Δ AIC two best models: 221,867; Fig 11). This shows the opposite to what we would expect if the colour distribution alone was the main source of compression: at the start, the distribution of colours in Place is highly non-uniform, as white tiles dominate the canvas, but as more colours are used the frequency distribution moves towards a uniform configuration (although it never becomes entirely uniform; global entropy for final time-step: 3.42 bit).

**Fig 12:**
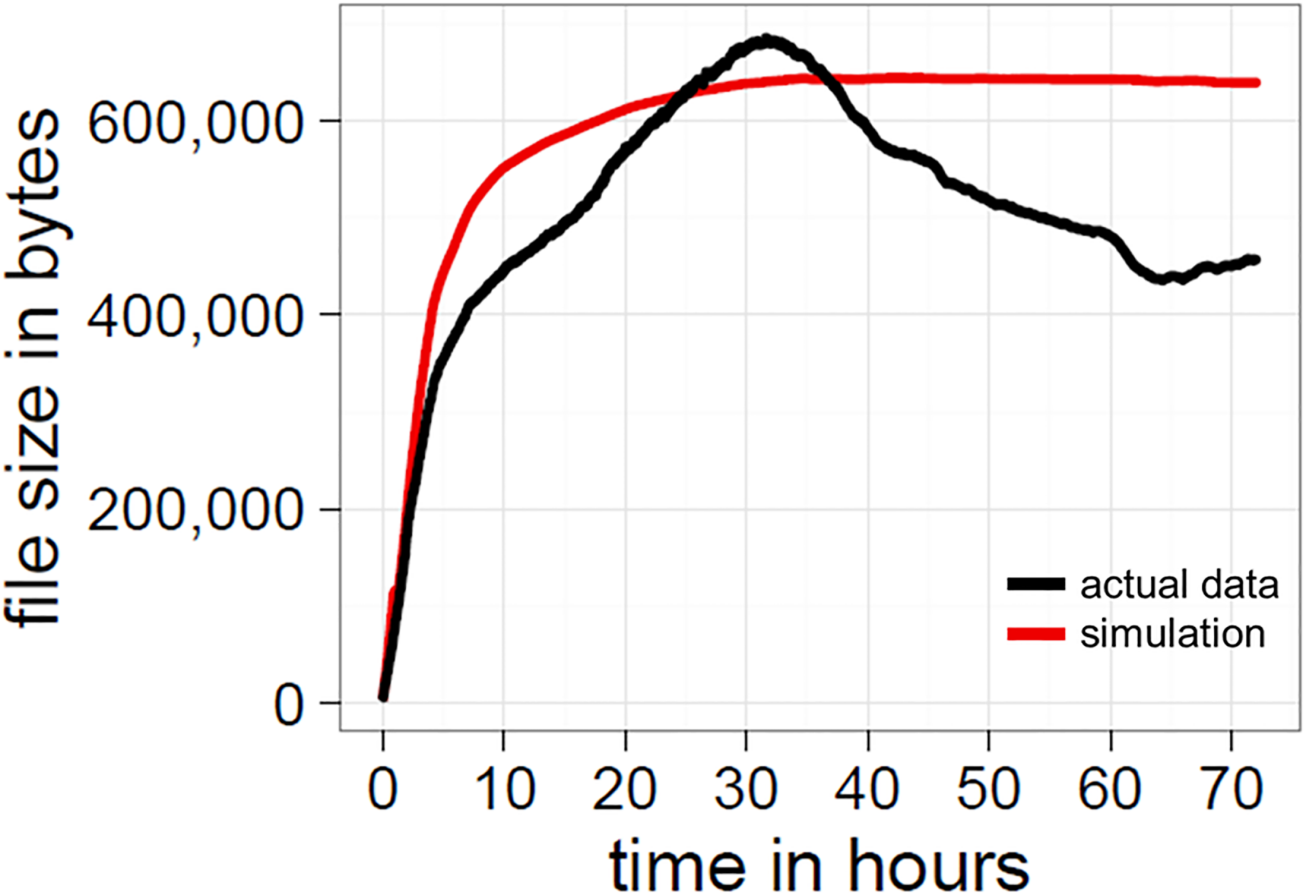
Comparison of compression between the actual data and the simulation. The black line represents the original file sizes and the red line the mean values for simulated images at each time step. For Place, the actual images are more compressible than randomly shuffled versions for a period during the first half and during the entire second half of the run-time.

Another possibility is that compression is being driven by the organisation of variation into structured, predictable patterns. We devised two tests for this. The first is to compare the actual compression data to simulations of randomly shuffled versions of the entire canvas, where the frequency distribution of coloured pixels is controlled for at each time-step (compare with Fig 4). Specifically, 100 shuffled images were created for each of the 259,194 time-steps, using the original colour frequencies of a specific time-step and assigning them to a randomly chosen position on a new 1000x1000 canvas. These randomly shuffled canvases were then compressed to .png files as described in the compression section. Randomly shuffling the canvas in this way allows us to preserve the frequency distribution of colours and remove the contribution of structure in making the canvas more compressible. This comparison reveals that early on, but, more importantly, also during the entire second half of the run-time of Place, the canvas was more compressible than random placement of the same colours (Fig 12). In fact, the compression outcomes in the simulations mirror the logarithmic trajectory of global entropy, confirming that the DEFLATE algorithm is also extracting structural regularities in pixel placements to increase compression (and not just relying on the frequency distribution of colours).

**Fig 13:**
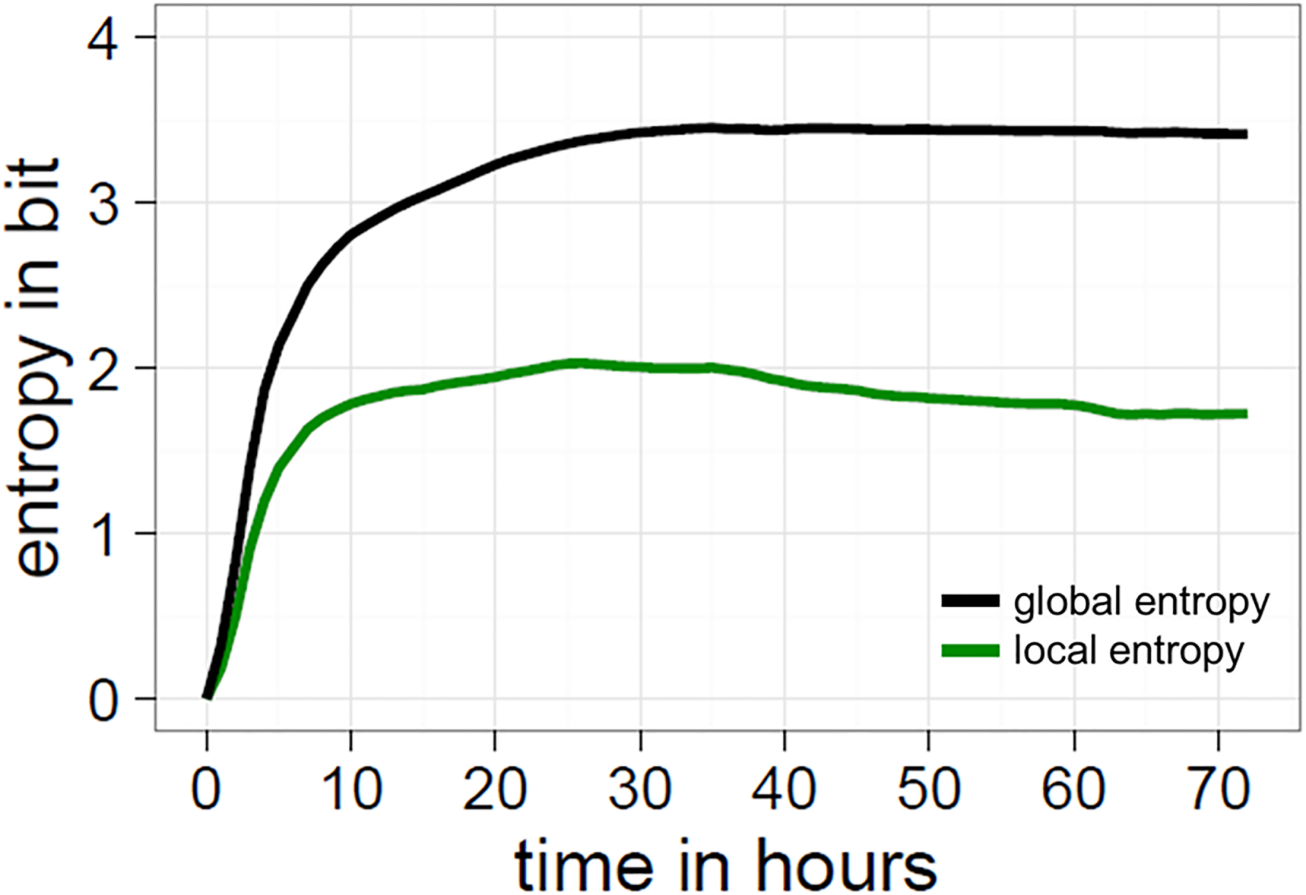
Comparison of summary entropy values both globally and locally. Whilst both types of entropy follow similar trajectories through time, global entropy (black line) remains much higher than local entropy (green line).

**Fig 14:**
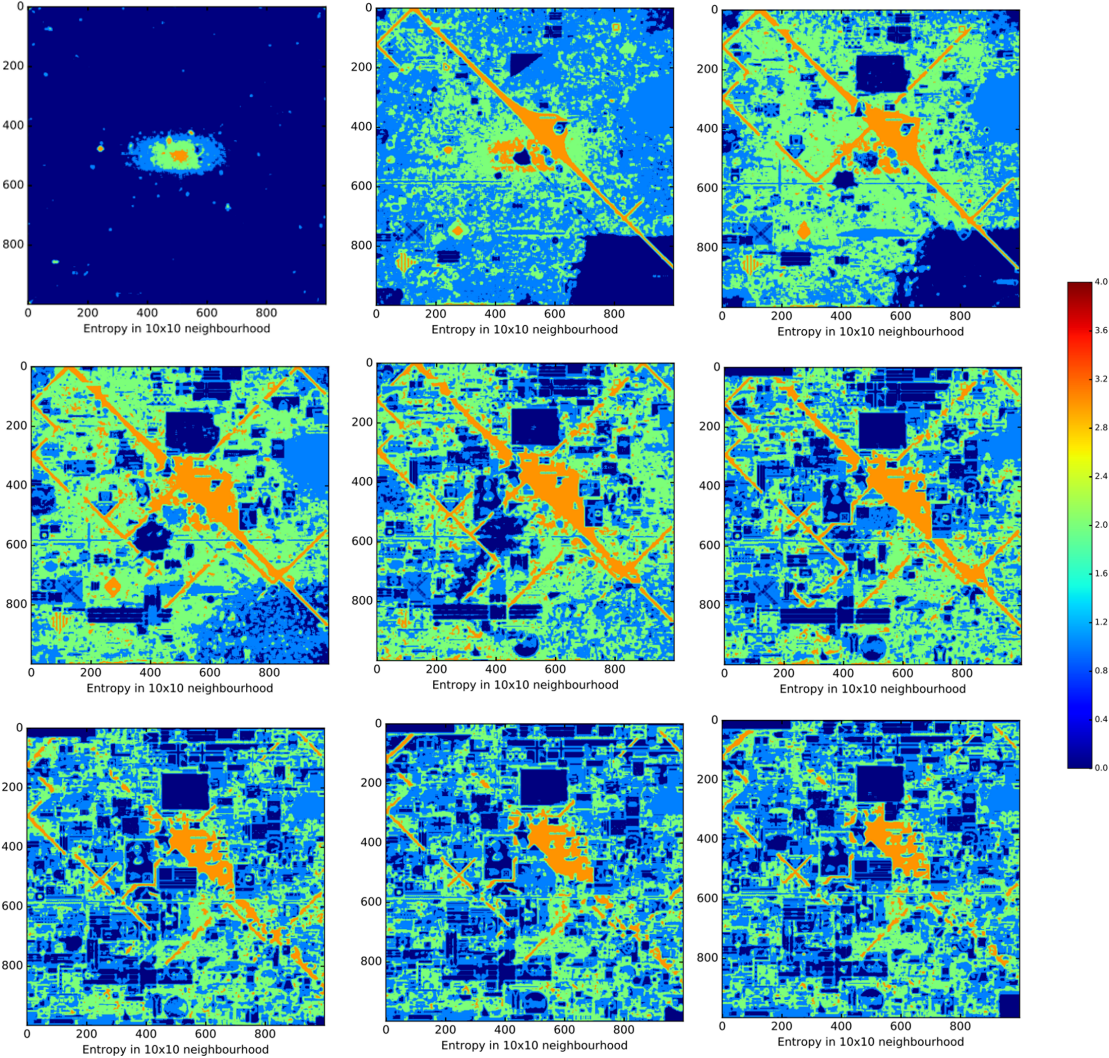
Heatmap of local entropy for nine time-stamps. Several heatmaps showing the local entropy for the following time-stamps (from top-left to bottom-right): 1-hour, 9-hours, 17-hours, 25-hours, 33-hours, 41-hours, 49-hours, 57-hours, 72-hours. The axis on the right shows the colour-coded entropy values (0 bit: dark blue and 4 bit: dark red). At 1-hour, the canvas is highly predictable at a local level, as the dark blue values indicate, and gradually becomes less predictable as time progresses. Between 25- and 41-hours Place reaches its highest levels of entropy locally (as highlighted by the large regions of orange and green), before gradually decreasing the entropy again (as the growing number of blue regions shows).

For our second test, we divided up the space into 10x10 pixel regions and measured the local entropy per pixel. Local entropy tells us whether the pixel placements within a given region are organised into more or less predictable frequency distributions. Contrasting our local and global measures of entropy therefore provides a proxy for discriminating between homogeneity and structuring processes. Homogeneity should show low global and low local entropy, whereas structure should show high global and low local entropy. At the early stages of Place local and global entropy are low, confirming our original expectation that compression is mainly being driven by the homogeneity of the space (i.e., white, unused pixels dominate). Yet, at the latter stages of Place, we find that global entropy is far higher than the local entropy (see Figs 13 and 14). Taken together, both the random simulation and local entropy show that the evolution of compressible patterns in the latter half of Place is mainly due to the underlying structure of artworks, and not simply the spreading of homogeneity.

### Does the canvas become more stable over time?

If stability is increasing, we should see this across three different time resolutions, with the signal becoming weaker as the resolution narrows (the ability to detect differences in stability diminishes due to the perfect retention of pixel placements between adjacent time-steps; see Methods for more details). Measuring stability in this way provides a robust measure for detecting a trend in the data. To test this hypothesis, we compared intercept-only models to linear models, and predicted that increasing slopes in the linear models outperform a simple intercept-only.

**Fig 15:**
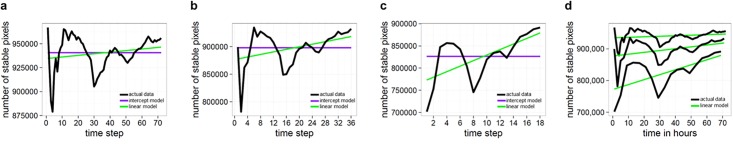
The development of stability over time. The black lines represent the number of stable pixels between two time slices, plotted for three different resolutions. Purple and green lines show intercept-only and linear models fitted to the data, respectively. (a) Number of stable pixels over time for 1-hour-intervals. (b) Number of stable pixels over time for 2-hour-intervals. (c) Number of stable pixels over time for 4-hour-intervals. (d) All three levels of granularity in one figure. There is a positive slope in the linear models for each, but the slope is steeper at coarser time intervals (as indicated by the lower numbers of stable pixels, more change can occur in these within a single time step).

As predicted, all model comparisons show a positive increase in the trajectory of stability (1-hour-slices: intercept only AIC: 1,609; linear AIC: 1,608; Δ AIC: 1; direct comparison: *F* = 3.11, *p* = .082; 2-hour-slices: intercept only AIC: 848; linear AIC: 844; Δ AIC: 4; direct comparison: *F* = 6.58, *p* = .015; 4-hour-slices: intercept only AIC: 445; linear AIC: 437; Δ AIC: 8; direct comparison: *F* = 11.20, *p* = .004; Fig 15a–15d). Although the effect in 1-hour-slices is marginal, this is consistent with our expectation that the signal becomes weaker as the time between slices decreases. Overall, these results show the latter stages of Place were less chaotic, suggesting participants were more likely to retain (and build upon) pre-existing artworks.

## Discussion

Using a novel, large-scale dataset, which links the interactions of more than one million individuals to the emergence of population-level pixel artworks, our results demonstrate the following: (i) That compression follows a predictable trajectory through time; (ii) Compression at the latter stages of Place is mainly driven by the canvas becoming more structured; (iii) The canvas becomes increasingly stable. From a non-structured state, where individual artworks exist relatively independently from one another, Place gradually transitions to a structured state where pixel placements form specialised, interdependent patterns. We provide three lines of evidence for this. First, the trajectories for variation and compression become decoupled at the latter stages of Place (as indexed by our random simulation results). Second, the canvas becomes increasingly stable, lending weight to the idea that pre-existing artworks are being maintained and extended. Lastly, the local regions of pixel placements remain low in entropy, whereas globally the entropy remains high, suggesting that variation is being maintained at a global level and organised into predictable distributions at a local level.

A major implication of our results is that the overall trajectory of compression is dependent on the density of the canvas. This is reflected in the non-linear time-course of compression: during the early phases, when the canvas is sparsely populated with artworks, Place becomes less compressed as more pixels are placed, yet it becomes more compressed at the latter stages when the canvas is densely saturated with artworks. Additional evidence is found in the direction of the interaction between activity and the number of used pixels: compression increases with a greater number of used pixels (i.e., a more densely populated canvas) *and* higher rates of activity (i.e., the number of pixels placed on the canvas at a specific point in time).

Cultural evolutionary dynamics can explain these population-level patterns in terms of competition and coexistence. Borrowing a simple explanatory concept from ecology, known as the *competitive exclusion principle* (i.e., two species competing for the same resource will result in the extinction of one competitor [37]), we argue that artworks must use different resources in order to coexist; otherwise competition takes place, and inevitably leads to the extinction of the losing artwork. In the case of Place, resources correspond to pixel positions, and competition takes place both within and between artworks via the placement of coloured pixels by users.

**Fig 16:**
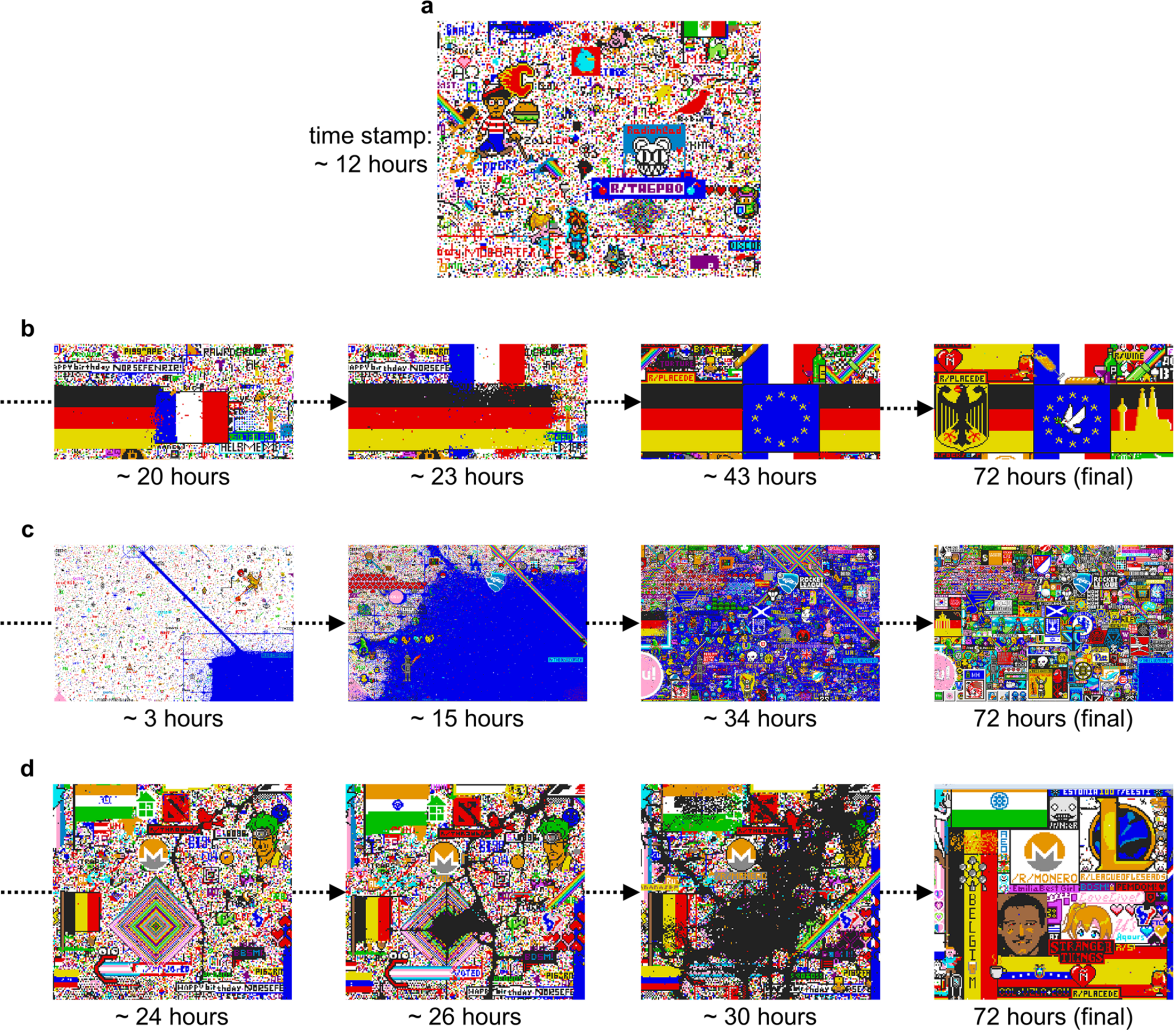
Selected examples in Place and their development over time. (a) Some isolated, simple and rather independent artworks in an early phase of the event. Note the chaotic single pixels and white space still dominating most of the space. (b) The development of the German-French (and later European) region. Germany naturally expanded its (horizontal) flag to the right, taking over French territory. The French (vertical) flag expanded to the top and bottom, and after diplomatic talks, a European alliance was formed; the two artworks became interdependent. Following the formation of this agreement, there was still constant modification within the two flags, adding national icons and sights. (c) The rise and fall of the Blue Corner. As one of the earliest groups, a subreddit formed that was determined to cover as much ground as possible in blue pixels, starting from the bottom right corner. This expansionary strategy was very successful early on, but later the group had to incorporate small artworks into their territory to be able to maintain it. Only a small portion of the Blue Corner remained upon completion of Place. (d) Destruction and innovation caused by the actions of the Void. Determined to destroy other creations, members of the Void group strategically attacked pieces of the canvas with black pixels. Ultimately, however, most of their efforts were overwritten over the time course of the event. Note that some, but not all of the original art destroyed by the Void resurfaces at the the end; some creations are entirely novel.

During the early phases, users have the capability to offset competition by creating artworks in unused regions, resulting in a canvas comprised of relatively idiosyncratic and isolated artworks (see Fig 16a). However, it is worth noting that compressible patterns are present at these early phases, as highlighted by the large regions occupied by Blue Corner and Green Lattice (especially around the 10-20 hour mark; Fig 16c). Such instances demonstrate that compressible patterns can emerge and spread, but importantly the overall canvas is still decreasing in compression. This is in contrast to the latter phases where the canvas follows a trajectory of increasing compression, even though it is densely saturated with a complex, interdependent ecosystem of artworks. We hypothesise that one consequence of fewer unused regions is that solutions to the competition-coexistence problem now amplify a preference for compressible patterns: artworks which are easier to produce, maintain, and generalise are favoured over other artworks. Compressible artworks are more amenable to preservation, as aspects of the artwork can be repaired and reproduced using a simple, generalisable rule. Having a generalisable rule also facilitates innovation: new artworks can be generated by incorporating regularities and patterns of one artwork into another.

Simplicity is not the only factor underpinning the survival of artworks, otherwise the canvas would be populated with homogeneous solutions such as Blue Corner (Fig 16c). Linking the low levels of homogeneity to the competition-coexistence problem is our second major finding: that increased compression is mainly driven by the canvas becoming more structured, integrated and specialised. By modifying pre-existing artworks to create new innovations, users are able to preserve elements of old artworks whilst facilitating the creation of new ones. We observe this in Fig 16b: here, the extension of old artworks injects structure into new ones, mitigating competition between artworks in a manner analogous to *niche differentiation* in ecology (i.e., artworks are able to coexist through integration and specialisation). Importantly, it is this spreading of structure which accounts for the increased compression in our results.

What remains unanswered is *why* Place becomes more structured as opposed to homogeneous. Arguably, structure seems to provide a more stable and less costly solution to the competition-coexistence problem than homogeneity. One reason for this is due to the diversity of the subreddit groups and their endogenous goals for creating artworks. We hypothesise that it is this diversity which results in diminishing returns for homogeneous artworks. To dominate the canvas, a homogeneous artwork would need to expend resources on growing as well as repairing damage to the artwork, with both factors minimising the competitive advantage of simplicity. In this sense, spreading homogeneity becomes increasingly costly because it requires large and well-organised groups to mitigate the countervailing effects of other groups (see Fig 16c and 16d). Had there been an overarching task for placing pixels, such as dominating as much space as possible, then it might have made homogeneous solutions more viable as groups could work together towards a common goal.

It is important to address the role played by white pixels in Place. One potential confound is that the initial state was a million white pixels and users could select white as a colour to place on the canvas. This raises the question as to whether or not the white space should be considered part of the culturally evolving artefact. We decided to fully include white as a colour in our analyses for the following reasons: First, it is unclear which cases of unused space were actually incorporated into artworks by users and which were simply not in use. Second, the potential confound is only really problematic during the first 30 hours of the runtime of Place, before the space is saturated with artworks. In contrast, our results mainly focus on the trajectories during latter phases of Place. Lastly, our predictions already factored in the effects of white pixels to some extent, which is why we formulated specific time-course trajectories for compression and variation in our models.

Another confound is in the use of the DEFLATE compression algorithm. In principle, randomness should act as an upper bound on the compression of image, yet we clearly see that our simulations produce randomly shuffled images which are more compressible than the original image (at approximately the 30-hour mark; see Fig 12). Such disparities suggests there is a bias in the way lossless compression algorithms exploit statistical regularities. It is already known that there are sequences with low algorithmic complexity which are not compressible by statistical estimators [44]. An example of this is the Thue-Morse sequence where a string is repeatedly appended by taking the Boolean complement of the current sequence, i.e., 0 becomes 01, 01 becomes 0110, and 0110 yields 01101001 etc. This provides a possible explanation as to why the compression algorithm might make the actual image less compressed than it appears. But it is also the case that randomly shuffled pixels will produce statistical regularities by chance. Randomly shuffling a non-uniform distribution of colours can create statistical regularities where there were previously none, with the detection and extraction of these regularities determined by the size of the sliding window used by the DEFLATE algorithm. It is possible that in our case the algorithm is both failing to capture some patterns in the actual image and compressing randomly generated regularities in the shuffled images.

Place provides a powerful model for investigating how cultural evolutionary processes can link spatial and temporal dynamics in producing predictable patterns. Future work is now well-placed to establish whether these findings can be reproduced and generalised to other cultural phenomena. Computational models [45] and mobile apps [46] provide two complementary routes for reproducing the scale of Place. In terms of generalisability, perhaps the most relevant comparison is to artistic traditions. Just as artworks are used to mark the identity of subreddits in Place, so too is art often employed to mark familial lineage via heraldic emblems [27] and group membership via graffiti tags [47]. Another point of comparison with graffiti is the collaborative nature of Place; a single artwork comprises the contributions of numerous individuals. Lastly, artistic traditions can also borrow from one another, as seen in the influence of Japanese woodblock prints on impressionist and post impressionist styles [48], which parallels the direct and indirect cross-fertilisation of ideas and styles in Place.

## Conclusion

Despite the cross-cultural heterogeneity of human societies, cultural evolutionary processes often deliver surprisingly convergent outcomes. We set out to investigate one of these proposed outcomes: the spread of compressible patterns. In particular, we used a novel, large-scale dataset to witness the *de novo* emergence of a culture in real-time: from an initially white canvas, where patterns of activity were idiosyncratic and independent, over a million individuals came together to produce a complex, interdependent ecosystem of artworks. Place not only demonstrates that the spread of compressible patterns is a signature of cultural evolution, it also highlights the interaction of temporal and spatial dynamics in determining the trajectories of change.
